# Environmental risk score of multiple pollutants for kidney damage among residents in vulnerable areas by occupational chemical exposure in Korea

**DOI:** 10.1007/s11356-024-33567-5

**Published:** 2024-05-14

**Authors:** Hyuna Jang, Kyung-Hwa Choi, Yong Min Cho, Dahee Han, Young Seoub Hong

**Affiliations:** 1 Department of Environmental Science, Baylor University, Waco, TX USA; 2 Department of Preventive Medicine, Dankook University College of Medicine, Cheonan, Republic of Korea; 3 Institute of Environmental Health, Seokyeong University, Seoul, Republic of Korea; 4 Department of Environmental Chemical Engineering, Seokyeong University, Seoul, Republic of Korea; 5 Department of Preventive Medicine, Dong-A University College of Medicine, Busan, Republic of Korea

**Keywords:** Multiple pollutants, Environmental risk score, Machine learning, Kidney damage, Occupational exposure

## Abstract

This study aimed to develop an environmental risk score (ERS) of multiple pollutants (MP) causing kidney damage (KD) in Korean residents near abandoned metal mines or smelters and evaluate the association between ERS and KD by a history of occupational chemical exposure (OCE). Exposure to MP, consisting of nine metals, four polycyclic aromatic hydrocarbons, and four volatile organic compounds, was measured as urinary metabolites. The study participants were recruited from the Forensic Research via Omics Markers (FROM) study (*n* = 256). Beta-2-microglobulin (β2-MG), N-acetyl-β-D-glucosaminidase (NAG), and estimated glomerular filtration rate (eGFR) were used as biomarkers of KD. Bayesian kernel machine regression (BKMR) was selected as the optimal ERS model with the best performance and stability of the predicted effect size among the elastic net, adaptive elastic net, weighted quantile sum regression, BKMR, Bayesian additive regression tree, and super learner model. Variable importance was estimated to evaluate the effects of metabolites on KD. When stratified with the history of OCE after adjusting for several confounding factors, the risks for KD were higher in the OCE group than those in the non-OCE group; the odds ratio (OR; 95% CI) for ERS in non-OCE and OCE groups were 2.97 (2.19, 4.02) and 6.43 (2.85, 14.5) for β2-MG, 1.37 (1.01, 1.86) and 4.16 (1.85, 9.39) for NAG, and 4.57 (3.37, 6.19) and 6.44 (2.85, 14.5) for eGFR, respectively. We found that the ERS stratified history of OCE was the most suitable for evaluating the association between MP and KD, and the risks were higher in the OCE group than those in the non-OCE group.

**Supplementary Information:**

The online version contains supplementary material available at 10.1007/s11356-024-33567-5.

## Introduction

Residents in environmentally vulnerable areas, located near industrial facilities, such as mines, smelters, and manufacturing facilities, have been chronically exposed to low concentrations of multiple pollutants (MP) (Herpin et al. 2009; Jo et al. 2021; Laney and Weissman 2014). Some residents may be involved in work that can expose them to MP. Occupational exposure has been implicated in various diseases because of its high concentration and persistent exposure (Massachusetts 2022; Paulin et al. 2015). Therefore, it is crucial to consider the association between MP and multiple health effects to assess the influence of chronic and persistent environmental hazards.

Environmental factors significantly contribute to the pathogenesis of chronic kidney disease (CKD) (Tsai et al. 2017). Heavy metals, such as lead (Pb), mercury (Hg), and cadmium (Cd), can cause renal proximal tubular damage and a decline in glomeruli (Kim et al. 2015). Polycyclic aromatic hydrocarbons (PAHs) may also contribute to kidney dysfunction by increasing oxidative stress (Farzan et al. 2016). The estimated glomerular filtration rate (eGFR) is generally accepted as the best measure of the overall kidney function (Ferguson and Waikar 2012). Sustained or chronically decreased eGFR is generally associated with a decrease in other renal functional parameters, resulting in an altered electrolyte and volume balance, decreased red blood cell production, hypertension, and altered bone mineral metabolism (Ferguson and Waikar 2012). The National Kidney Foundation Kidney Disease Outcomes Quality Initiative defines the stages of CKD based on eGFR (Ferguson and Waikar 2012). Before developing the eGFR marker, β2-microglobulin (β2-MG) and N-acetyl glucosaminidase (NAG) were used as biomarkers for renal microtubular damage. If the reabsorption rate of the renal tubule decreases, the amount of protein and enzyme in the urine increases, and the concentrations of biomarkers increase (Satarug et al. 2010).

Environmental risk score (ERS) is a useful tool to summarize the risk of exposure to MP in environmental epidemiological research. One previous study constructed the ERS of 149 environmental pollutants, including heavy metals, phthalates, PAHs, and dioxins (Park et al. 2014). It is possible to characterize the ERS of MP even for high degrees of correlation or high-dimensional data using machine learning methods (Fu et al. 2022; Park et al. 2017; Wang et al. 2018). Machine learning methods can improve model performance against statistical challenges, such as collinearity or failure of fitting. Several studies have reported the constructed ERS of various exposure biomarkers and health outcomes and have measured performance. Five helpful methods can be used to construct MP models of phthalates, phenols, pesticides, perchlorate, and related anions (Sun et al. 2013); the ERS of heavy metals for cardiovascular disease (Park et al. 2017); ERS of heavy metal mixture for obesity (Wang et al. 2018); ERS of 24 urinary metals for heart rate (Fu et al. 2022); ERS of 24 metals for eGFR (Rodriguez-Villamizar et al. 2023). Therefore, an ERS of MP, including metals, PAHs, and VOCs, for kidney damage (KD) in environmentally vulnerable residents remains to be constructed.

This study aims to develop the ERS of the MPs that cause KD in residents of environmentally vulnerable areas in Korea and evaluate the association between ERS and KD caused by occupational chemical exposure (OCE).

## Material and methods

### Study population and participants

The cross-sectional study, Forensic Research via Omics Markers (FROM), is being conducted in Korea (2021–2025) to develop biomarkers for assessing health effects and tracking diseases by exploring environmental pollutants that reflect the characteristics of vulnerable areas using biological samples. The FROM study selected survey areas classified as environmentally vulnerable regions by the Korean central and local governments (Choi et al. 2023). The central and local governments have conducted investigations to monitor or determine the health effects of residents due to environmental exposure from sources, such as abandoned metal mines, smelters, and industrial complexes. Recruited participants were residents or from another survey by the local or central government; they voluntarily agreed to participate after being given a sufficient explanation of the objectives of the study. A total of 298 individuals (263 from vulnerable areas and 35 from the control area) were recruited for the FROM study. Among the 298 participants, 256 individuals with normal creatinine concentrations (> 30 mg/dL and < 300 mg/dL) were selected for the present study.

**Fig. 1:**
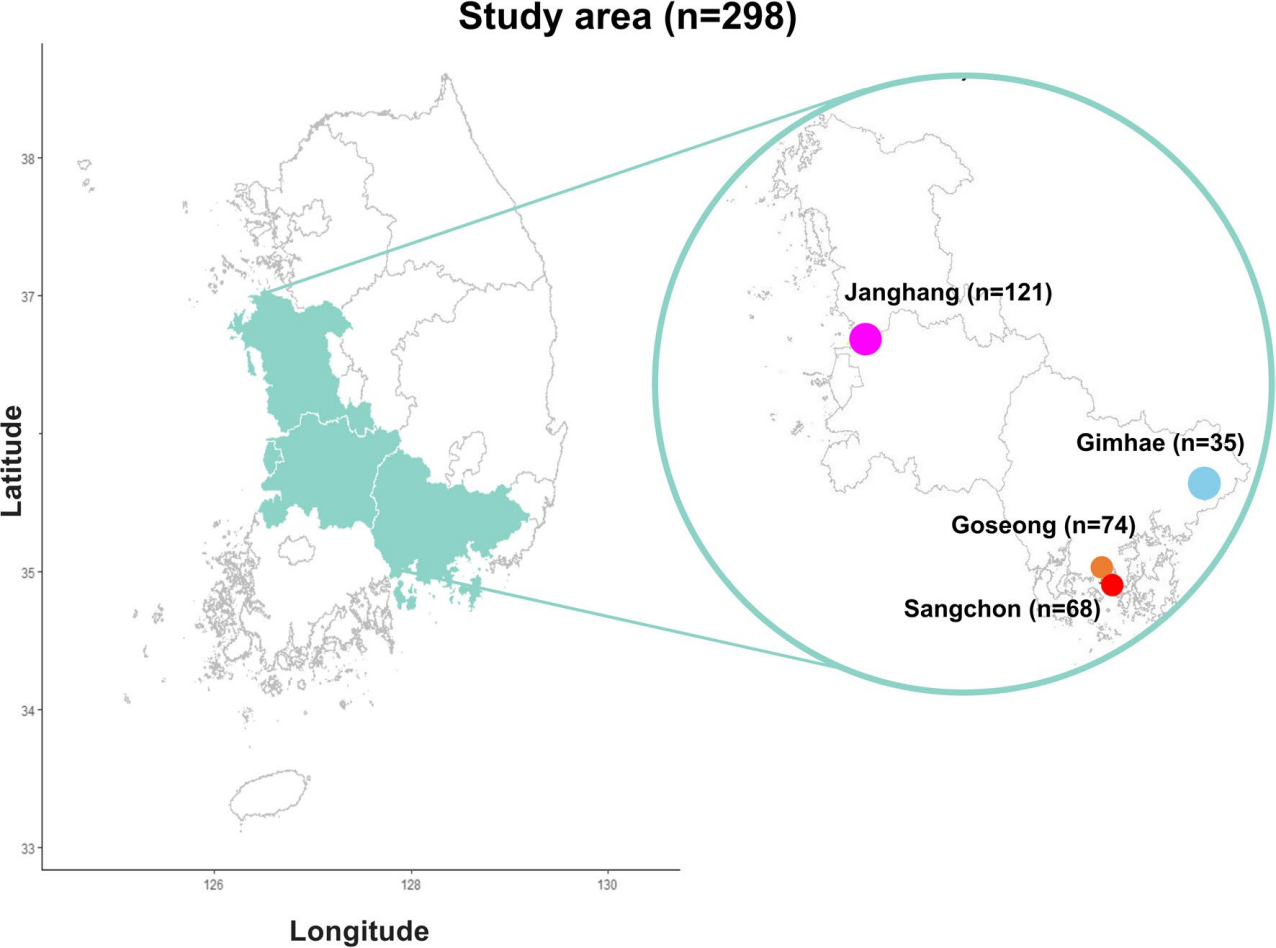
Map of study areas as a part of the Forensic Research via Omics Marker (FROM) study in Korea. Goseong and Sangchon are exposure areas around abandoned metal mines, Janghang is an exposure area around the smelter, and Gimhae is the control area

Our study was conducted in the following four study areas (Fig. 1): Goseong and Sangchon as the exposure areas around abandoned metal mines, Janghang as the exposure area around the smelter, and Gimhae as the control area. In Goseong and Sangchon, the problem of Cd poisoning was first raised in 2004 (Kwon 2011). The health effects survey discovered that it was caused by the contamination of living environments, such as crops and drinking water, by pollutants from abandoned metal mines (Kwon 2011). The participants in Goseong and Sangchon lived within 1.18–3.76 km and 4.09–5.63 km from the source of pollution. Janghang is situated around a copper smelter that was operational from 1930 to 1989 (Kim et al. 2016). The smelter was built in the 1930s and led to Korean industrialization during the 1960s and 70 s. Since 1989, serious heavy metal contamination has been found in crops and soil, and residents have suffered from Cd poisoning for decades (Kim et al. 2016). The participants in Janghang lived within 1.33–23.60 km from the source of pollution. Gimhae was used as the control area.

In this study, personal and health data were collected using a self-reported questionnaire from July 25 to October 26, 2021. The survey was conducted in person by survey assistants previously trained to ask questions to elderly participants. This study was approved by the DONGA University Institutional Review Board of Korea (2–1,040,709-AB-N-01–202105-BR-002–08). Informed consent was obtained from all participants, and the study followed the guidelines of the Declaration of Helsinki for research on human participants.

### Metabolites

Pollutant concentrations were measured in the urine samples of the study population. The following seventeen metabolites were analyzed: nine metals (Hg, vanadium [V], chromium [Cr], manganese [Mn], nickel [Ni], molybdenum [Mo], Cd, antimony [Sb], and Pb); four PAHs (2-naphthol [2-naph], 1-hydroxypyrene [1-ohp], 2-hydroxyfluorene [2-ohf], and 1-hydroxyphenanthrene [1-ohph]); and four volatile organic compounds (VOCs) (phosphoglyceric acid [PGA]; trans, trans-muconic acid [t,t-MA]; methylhippuric acid [MHA]; and benzylmercapturic acid [BMA]). Urine samples were collected and stored at − 80 ℃ until analysis. The urine samples were analyzed for metals in March 2022 and for the other compounds within 1 month of collection. A previous study reported that urinary concentrations of As, Cd, Pb, Tl, and Zn stored at − 80 °C remained stable for more than a decade (Beauval et al. 2022). Hg in the urine samples was analyzed by adding 0.1 mL of the sample to a mercury analyzer (MA-3000, NIC, Japan) after the sample was thawed and thoroughly mixed using a vortex mixer. All metals, except Hg, were analyzed using inductively coupled plasma-mass spectrometry (Agilent 7800, Agilent, USA) after diluting 0.3 mL of urine sample with 2% nitric acid (2.7 mL). We replaced the concentrations below the limit of detection (LOD) with LOD/2. The concentrations of these metabolites were adjusted for measuring urinary creatinine levels.

### Biomarkers for kidney damage (KD)

β2-MG, NAG, and eGFR were used as biomarkers of KD. β2-MG and NAG were used as biomarkers for renal microtubular damage. β2-MG and NAG levels of > 300 μg/L (Kawai et al. 2010) and > 11.5 IU/L (Park 2020), respectively, indicate acute kidney injury (AKI). The samples were analyzed using an automated chemistry analyzer (Cobas 702, Roche Diagnostics System, Switzerland) with a wavelength of 700 nm. Standard reagents from Roche Diagnostics System and Nittobo Medical Co. were used for β2-MG and NAG, respectively. After the samples were thawed and thoroughly mixed using a vortex mixer, 0.5 mL of the sample was directly added to the analyzer. The concentrations were adjusted for creatinine (μg/L Crea or IU/L Crea.).

The eGFR is a marker of CKD. If the value of eGFR is $<$ 90, it indicates mild KD (Ferguson and Waikar 2012). eGFR was calculated using the following equation:

$$\text{GFR}\hspace{0.17em}=\hspace{0.17em}141\times{\text{min}(S_{cr}/\kappa,1)}^\alpha\times{\text{max}(S_{cr}/\kappa,1)}^{-1.209}\times{0.993}^{Age}\times1.018\lbrack\mathrm{if}\;\mathrm{female}\rbrack$$,

 where ${S}_{cr}$ is the serum creatinine in mg/dL, $\kappa$ is 0.7 for females and 0.9 for males, $\alpha$ is − 0.329 for females and − 0.411 for males, min indicates the minimum of Scr/κ or 1, and max indicates the maximum of Scr/κ or 1 (National Institutes of Health 2022).

### Covariates

Covariate data were obtained from the FROM survey and included the sex, age group (29–<65 years, 65–74 years, and $\ge$ 75 years), medication for any disease (No and Yes), study area (Gimhae, Goseong, Sangchon, and Janghang), tertile of residence period (cutoff points 12.92 and 50.00 years), tertile of urinary cotinine level (cutoff points 2.09 and 6.97 μg/g Crea.), and the period of OCE classified by the median (None, < 4.5 years, or 4.5 – ≤ 60 years). A history of OCE was defined as being engaged in one of 15 occupations, such as mining, paint manufacturing/painting, battery manufacturing, welding, smelting/alloying, wire/cable manufacturing, plating, printing industry, gas station, fluorescent lamp manufacturing, rubber/PVC/plastic manufacturing, pesticide/insecticide manufacturing, radiation/radiation shielding, plastic coloring agent/pigment work, and weaving work.

### Statistical analyses

Data pertaining to the general characteristics of participants are presented as frequency and proportion, geometric mean (GM), and the 95% confidence interval (CI) according to each biomarker. To compare the GM among the general characteristics, t-test or ANOVA were performed after log-transformation. Pairwise Spearman correlations were calculated for the seventeen metabolites, and the results are displayed in a correlation matrix heat map.

### Selection of optimal ERS model for each biomarker in vulnerable areas

**Fig. 2:**
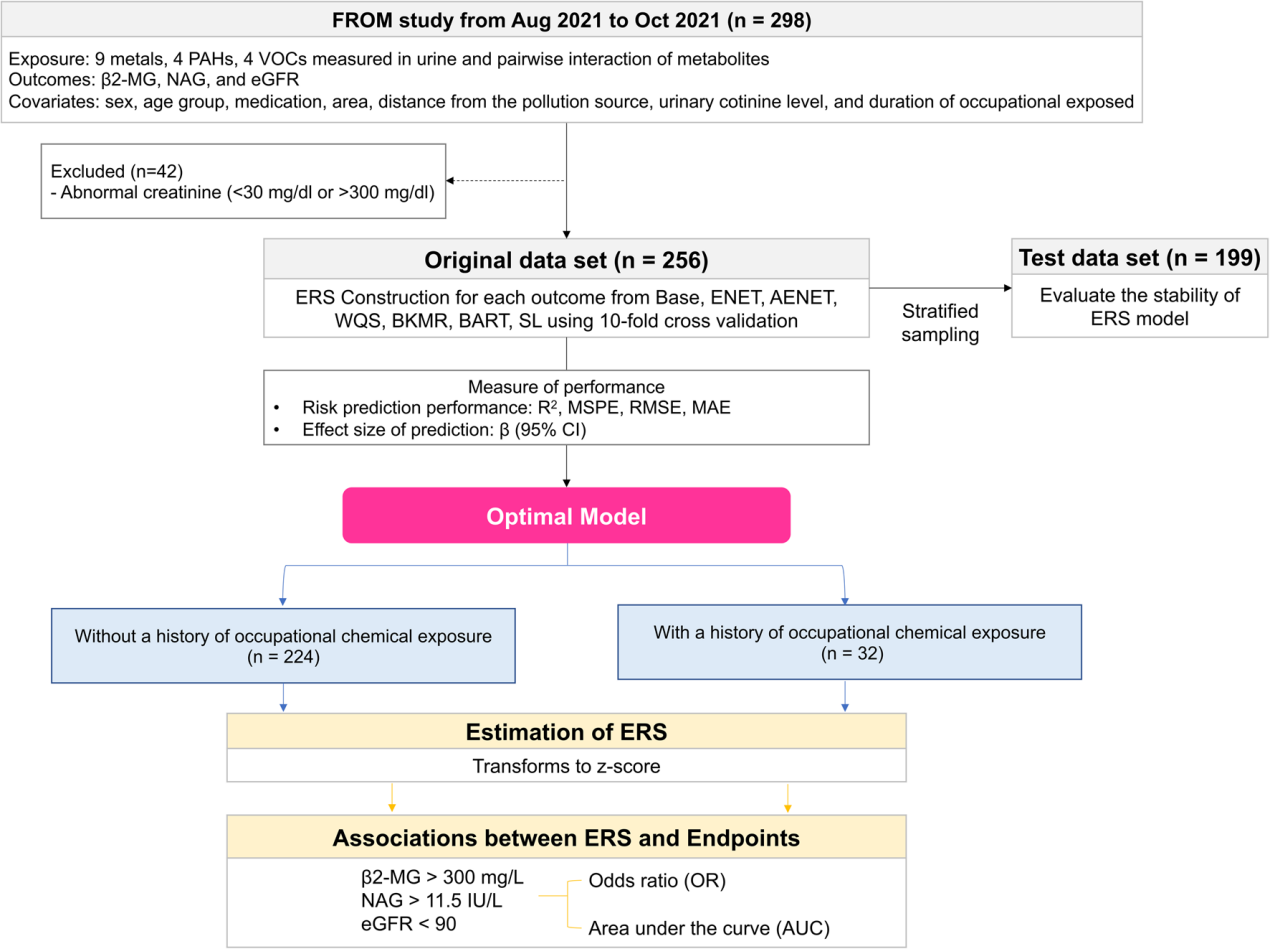
Process of construction of the environmental risk score (ERS). The following seventeen metabolites were analyzed: nine heavy metals in urine (Hg, V, Cr, Mn, Ni, Mo, Cd, Sb, and Pb), four polycyclic aromatic hydrocarbons (PAHs) (2-naphthol [2-naph], 1-hydroxypyrene [1-ohp], 2-hydroxyfluorene [2-ohf], and 1-hydroxyphenanthrene [1-ohph]), and four volatile organic compounds (VOCs) (phosphoglyceric acid [PGA], trans, trans-muconic acid [t,t-MA], methylhippuric acid [MHA], and benzylmercapturic acid [BMA]). β2-MG, beta-2-microglobulin; NAG, N-acetylglucosaminidase; eGFR, estimate glomerular filtration rate; base model is multiple linear regression; ENET, elastic net; AENET, adaptive elastic net; WQS, weighted quantile sum regression; BKMR, Bayesian kernel machine regression; BART, Bayesian additive regression tree; SL, super learner. Performance measures are MSPE, mean-squared-prediction error; RMSE, root-mean-squared error; and MAE, mean absolute error

We used original and test datasets to evaluate the stability of ERS algorithms. The test dataset was sampled using stratified sampling from the original dataset, with consideration of all covariates and a sampling ratio of approximately 80% (*n* = 199) and a tenfold cross-validation process to stabilize the results (Mueller et al. 2016). Figure 2 shows the algorithm. To explore the optimal ERS model of residents in vulnerable areas for each biomarker, we considered each metabolite and pairwise interaction of metabolites for exposure (Park et al. 2017) and applied multiple regression as the base model and used the following six statistical models (Park et al. 2017): linear model elastic net (ENET), adaptive elastic net (AENET), weighted quantile sum regression (WQS), Bayesian kernel machine regression (BKMR), Bayesian additive regression tree (BART), and super learner (SL). All variables were base-10 logarithm transformed and scaled to compare the model performance. The six models were fitted with both crude and adjusted models, including all covariates except for OCE, which was used for stratified analysis, and model selection was based on the adjusted model. Finding the tuning parameter and methods of cross-validation followed Park et al. (2017) for ENET, AENET, BKMR, BART, and SL and Fu et al. (2022) and Tanner et al. (2019) for WQS. Optimal models for each biomarker were selected by comparing their performances. The performance measures were *R*^2^, mean-squared-prediction error (MSPE), root-mean-square error (RMSE), and mean absolute error (MAE) for risk prediction performance, and *β* (95% CI) for confirming the stability of effect size of prediction. The R packages are displayed in Table A1 (Bobb 2022; Polley et al. 2021; Hastie 2022; Chipman and McCulloch 2016; Stekhoven and Bühlmann 2012; Kapelner and Bleich 2016; Kuhn 2022; Meyer et al. 2022; Peters and Hothorn 2022; Renzetti et al. 2021; Simon et al. 2011; Chen et al. 2022; Weston and Wickham 2014; Yang et al. 2022).

To measure the effect size between each pollutant and the biomarkers of KD, we estimated coefficients for ENET, AENET, and WQS, used posterior inclusion probability (PIP) for BKMR and BART, and calculated variable importance (VI) for SL as follows:

$$\mathrm{VI }= (SSE-SSE\left(-i\right)) / SSE$$,

 where ${\text{SSE}}$ is the sum of the squared error from a fitting model with all exposures, and ${\text{SSE}}(-{\text{i}})$ is the sum of squared error from a fitting model that removes one metabolite (Park et al. 2017).

These methods have been frequently used by researchers to estimate the effects of mixed compounds on health and evaluate the best performance of models in environmental epidemiology (Fu et al. 2022; Park et al. 2017; Sun et al. 2013; Wang et al. 2018; Weng et al. 2022).

### Estimation of ERS stratified based on the history of occupational chemical exposure

The selected models were used to estimate the ERS stratified based on the history of OCE (Fig. 2). We also described the stratified GM and 95% CI of urinary metabolite according to the history of OCE. The ERS, according to the history of OCE, was constructed using the optimal model and adjusted for all covariates. We represented the distribution of the estimated ERS using boxplots. Odds ratios (ORs) were calculated to evaluate the risk of kidney damage on estimated ERS using simple logistic regression. In addition, we calculated the sensitivity, specificity, cutoff value of ERS, and the area under the receiver operating characteristic (ROC) curve (AUC) to measure the accuracy of each model. ERSs were transformed to *z*-scores, indicating how far the value is from the mean, and kidney damage was measured using the dichotomous variable of urinary biomarkers.

## Results

Table 1 shows the distribution of KD markers based on the general characteristics of 256 residents: 157 females (61.3%) and 99 males (38.7%). Most individuals were aged above 65 years, with 29.3% being smokers and 12.5% having a history of OCE. The concentration of β2-MG showed an increasing trend with medication for any disease (*p* = 0.03), history of OCE (*p* = 0.03), and period of OCE (*p* = 0.01), and eGFR and age (*p* < 0.0001), whereas it exhibited a decreasing trend with smoking history (*p* < 0.0001), history of OCE (*p* < 0.0001), and period of OCE (*p* < 0.0001). However, no significant differences were observed between the other general characteristics and KD markers. The concentration of urinary metabolites exhibited high correlations among PAHs and metals (Fig A1).

**Table 1:** General characteristic and distribution of each kidney damage marker

	*N* (%)	β2-microglobulin (µg/L Crea.)			N-acetylglucosaminidase (IU/L Crea.)			Estimated glomerular filtration rate (eGFR)^a^		
		GM	95% CI	*p* value	GM	95% CI	*p* value	GM	95% CI	*p* value
All	256 (100)	157.0	(136.51, 180.45)		4.9	(4.05, 5.81)		26.7	(25.07, 28.44)	
Gender				0.59			0.99			< 0.0001
Male	99 (38.7)	149.6	(116.71, 191.75)		4.9	(4.13, 5.69)		14.1	(13.90, 14.33)	
Female	157 (61.3)	161.8	(136.87, 191.22)		4.9	(3.67, 6.41)		39.9	(39.47, 40.36)	
Age (years)				0.10			0.42			0.99
29–< 65	69 (27.0)	129.9	(105.13, 160.56)		3.9	(3.21, 4.68)		26.3	(23.10, 29.85)	
65–74	94 (36.7)	161.7	(131.62, 198.66)		5.7	(5.04, 6.55)		27.4	(24.66, 30.41)	
75+	93 (36.3)	175.2	(131.66, 233.21)		4.8	(3.04, 7.65)		26.4	(23.77, 29.22)	
Co-morbidity (hypertension or diabetes)				0.12			0.82			0.75
No	117 (45.7)	139.2	(116.99, 165.55)		5.0	(4.28, 5.75)		27.0	(24.53, 29.72)	
Yes	139 (54.3)	173.7	(140.51, 214.67)		4.8	(3.49, 6.49)		26.5	(24.31, 28.79)	
Medication for any disease				0.03			0.74			0.81
No	49 (19.1)	114.6	(89.72, 146.32)		4.6	(3.64, 5.71)		26.3	(22.44, 30.77)	
Yes	207 (80.9)	169.1	(143.85, 198.77)		4.9	(3.96, 6.12)		26.8	(25.01, 28.72)	
Area^b^				0.20			0.66			0.39
Gimhae	32 (12.5)	144.7	(97.92, 213.78)		4.9	(4.02, 6.05)		28.8	(23.98, 34.52)	
Goseong	68 (26.6)	123.6	(93.50, 163.34)		6.5	(5.50, 7.65)		27.1	(24.03, 30.64)	
Sangchon	63 (24.6)	201.1	(145.91, 277.03)		3.0	(1.54, 5.88)		25.9	(22.72, 29.60)	
Janghang	93 (36.3)	162.6	(132.03, 200.13)		5.4	(4.65, 6.24)		26.2	(23.57, 29.20)	
Period of residence (years)				0.63			0.81			0.12
T1 (< 12.92)	85 (33.2)	147.4	(113.76, 191.03)		4.5	(3.76, 5.45)		24.0	(21.41, 26.97)	
T2 (12.92–< 50.00)	80 (31.3)	163.8	(135.26, 198.29)		5.3	(4.65, 6.02)		29.2	(26.22, 32.58)	
T3 (50.00–93.00)	91 (35.6)	160.3	(122.80, 209.30)		4.8	(2.99, 7.66)		27.2	(24.53, 30.20)	
Smoking history				0.76			0.74			< 0.0001
No	181 (70.7)	154.8	(131.89, 181.63)		4.8	(3.72, 6.07)		34.0	(32.07, 35.94)	
Yes	75 (29.3)	162.3	(122.11, 215.81)		5.1	(4.23, 6.12)		15.0	(14.16, 15.80)	
Urinary cotinine level (μg/g Crea.)				0.12			0.82			0.29
T1 (< 2.09)	85 (33.2)	150.8	(122.32, 185.95)		5.6	(4.93, 6.42)		28.0	(25.07, 31.15)	
T2 (2.09– < 6.97)	85 (33.2)	129.5	(105.41, 159.01)		3.8	(2.31, 6.26)		26.5	(23.74, 29.57)	
T3 (6.97–3851.99)	86 (33.6)	197.5	(146.57, 266.08)		5.3	(4.45, 6.39)		25.7	(22.97, 28.80)	
History of occupational chemical exposure				0.03			0.42			< 0.0001
No	224 (87.5)	148.3	(128.94, 170.54)		4.7	(3.84, 5.78)		28.2	(26.39, 30.10)	
Yes	32 (12.5)	233.5	(135.62, 402.16)		5.9	(4.67, 7.47)		18.3	(15.59, 21.48)	
Period of occupational chemical exposure (years)				0.01			0.33			< 0.0001
None	224 (87.50)	148.3	(128.94, 170.54)		4.7	(3.84, 5.78)		28.2	(26.39, 30.10)	
< 4.5	16 (6.3)	154.6	(89.69, 266.56)		5.0	(3.68, 6.85)		18.5	(14.57, 23.43)	
4.5– ≤ 60	16 (6.3)	352.7	(134.19, 927.11)		6.9	(4.80, 10.04)		18.1	(14.20, 23.13)	

*GM*, geometric mean; *CI*, confidence interval

*p* value was estimated using t-test or ANOVA after log-transformation

^a^GFR = 141 × min (S_cr_/κ, 1)^α^ × max (S_cr_/κ, 1)^−1.209^ × 0.993^Age^ × 1.018 [if female], where S_cr_ is serum creatinine in mg/dL, *κ* is 0.7 for females and 0.9 for males, *α* is − 0.329 for females and − 0.411 for males, min indicates the minimum of Scr/κ or 1, and max indicates the maximum of Scr/κ or 1

^b^The pollution sources of Goseong and Sangchon are abandoned metal mines, and that of Janghang is a smelter. The participants in Goseong, Sangchon, and Janghang lived within distances of 1.18 to 3.76 km, 4.09 to 5.63 km, and 1.33 to 23.60 km from the source of pollution

We assessed the model reliability using two datasets (Table A2 and Fig A2). Table 2 displays the performance of each model adjusted for all covariates. The performances of all models were similar for the original and test datasets. Among the six models applied, BKMR demonstrated superior predictive performance with the highest *R*^2^ (β2-MG 0.98, NAG 0.99, and eGFR 0.79) and lowest MSPE (β2-MG 0.07, NAG 0.01, and eGFR 0.24), RMSE (β2-MG 0.26, NAG 0.12, and eGFR 0.49), and MAE (β2-MG 0.19, NAG 0.10, and eGFR 0.38) in the original dataset. The coefficients were stable across datasets and estimated for the original and test datasets at 1.28 and 1.16, 1.33 and 1.08, and 0.17 and 0.13 for β2-MG, NAG, and eGFR, respectively. Table A3 shows the performance of each model without the covariates (crude model). Thus, we selected BKMR as the optimal model for all markers. According to datasets and models, the top three most effective metabolites for β2-MG were identical in ENET, AENET, BKMR, and BART and similar for NAG and eGFR. However, there were slight differences in the results from SL (Fig A3).

**Table 2:** Comparison among the models of the distribution and performance of environmental risk score (ERS) of urinary metabolites for biomarkers by kidney damage markers

Model	Base			ENET				AENET				WQS				BKMR			BART		SL	
Data	Original	Test		Original		Test		Original		Test		Original		Test		Original		Test	Original	Test	Original	Test
β2-microglobulin (μg/dL)																						
Distribution of ERS																						
Mean (SD)	0.01 (0.86)	− 0.02 (0.96)		0.01 (0.35)		− 0.02 (0.33)		0.01 (0.38)		− 0.02 (0.45)		0.01 (0.46)		− 0.02 (0.52)		0.00 (0.77)		− 0.02 (0.87)	0.01 (0.28)	0.01 (0.28)	− 0.01 (0.41)	0.01 (0.44)
Range (Min, Max)	(− 2.41, 3.14)	(− 2.94, 3.68)		(− 0.79, 1.14)		(− 0.78, 1.21)		(− 0.91, 0.99)		(− 1.03, 1.54)		(− 0.95, 1.57)		(− 1.12, 1.77)		(− 2.18, 3.28)		(− 2.55, 3.37)	(− 0.55, 0.82)	(− 0.62, 0.63)	(− 1.29, 1.26)	(− 1.19, 1.06)
Risk prediction performance																						
R^2^	0.75	0.90		0.25		0.26		0.24		0.30		0.22		0.27		*0.98*		*0.99*	0.35	0.36	0.08	0.07
MSPE	0.25	0.10		0.75		0.79		0.75		0.72		0.76		0.75		*0.07*		*0.03*	0.73	0.76	0.92	0.99
RMSE	0.50	0.32		0.87		0.89		0.87		0.85		0.87		0.86		*0.26*		*0.16*	0.85	0.87	0.96	0.99
MAE	0.38	0.23		0.62		0.64		0.63		0.63		0.67		0.67		*0.19*		*0.12*	0.61	0.62	0.70	0.72
Effect size of prediction																						
$\hspace{1em}\beta$ (95% CI)	1.00 (0.93, 1.07)	1.00 (0.95, 1.05)		1.42 (1.11, 1.72)		1.56 (1.18, 1.93)		1.28 (1.00, 1.57)		1.24 (0.97, 1.50)		1.00 (0.76, 1.24)		1.00 (0.76, 1.24)		1.28 (1.26, 1.30)		1.16 (1.15, 1.18)	2.10 (1.75, 2.46)	2.21 (1.80, 2.63)	0.67 (0.38, 0.96)	0.59 (0.27, 0.92)
N-acetylglucosaminidase (IU/L)																						
Distribution of ERS																						
Mean (SD)	0.06 (0.39)		0.08 (0.44)		0.06 (0.16)		0.08 (0.19)		0.06 (0.17)		0.08 (0.21)		0.06 (0.24)		0.08 (0.26)		0.05 (0.34)	0.08 (0.43)	0.00 (0.10)	0.00 (0.11)	0.00 (0.43)	0.00 (0.34)
Range (Min, Max)	(− 1.15, 1.19)		(− 1.15, 1.21)		(− 0.40, 0.55)		(− 0.47, 0.67)		(− 0.40, 0.62)		(− 0.58, 0.83)		(− 0.64, 0.60)		(− 0.67, 0.74)		(− 0.83, 0.92)	(− 1.05, 1.14)	(− 0.26, 0.27)	(− 0.25, 0.30)	(− 1.04, 1.58)	(− 0.84, 0.84)
Risk prediction performance																						
R^2^	0.74		0.91		0.26		0.32		0.25		0.33		0.28		0.32		*0.99*	*1.00*	0.22	0.27	0.05	0.06
MSPE	0.05		0.02		0.15		0.15		0.16		0.15		0.15		0.15		*0.01*	*0.00*	0.18	0.18	0.30	0.25
RMSE	0.23		0.14		0.39		0.39		0.39		0.38		0.38		0.38		*0.12*	*0.04*	0.42	0.42	0.55	0.50
MAE	0.18		0.11		0.30		0.30		0.30		0.30		0.30		0.30		*0.10*	*0.03*	0.33	0.33	0.43	0.39
Effect size of prediction																						
$\hspace{1em}\beta$ (95% CI)	1.00 (0.93, 1.07)		1.00 (0.96, 1.04)		1.41 (1.11, 1.70)		1.41(1.12, 1.70)		1.33 (1.04, 1.62)		1.23 (0.98, 1.49)		1.00 (0.80, 1.20)		1.00 (0.79, 1.21)		1.33 (1.32, 1.35)	1.08 (1.07, 1.08)	2.13 (1.62, 2.64)	2.24 (1.71, 2.76)	0.24 (0.11, 0.37)	0.34 (0.15, 0.53)
Estimated glomerular filtration rate (eGFR)																						
Distribution of ERS																						
Mean (SD)	− 0.02 (0.85)	− 0.01 (0.94)		− 0.02 (0.53)		− 0.01 (0.58)		− 0.02 (0.54)		− 0.01 (0.49)		− 0.02 (0.59)		− 0.01 (0.65)		− 0.03 (0.66)		− 0.01 (0.72)	0.00 (0.26)	0.00 (0.16)	0.00 (0.24)	− 0.02 (0.30)
Range (Min, Max)	(− 2.12, 2.66)	(− 2.29, 2.96)		(− 1.54, 1.28)		(− 1.47, 1.58)		(− 1.54, 1.31)		(− 1.19, 1.19)		(− 1.43, 1.41)		(− 1.55, 1.73)		(− 1.71, 1.99)		(− 1.72, 2.23)	(− 0.58, 0.88)	(− 0.41, 0.51)	(− 0.69, 0.81)	(− 0.69, 1.06)
Risk prediction performance																						
R^2^	0.78	0.90		0.44		0.48		0.44		0.44		0.37		0.43		*0.79*		*0.91*	0.29	0.22	0.02	0.00
MSPE	0.21	0.10		0.54		0.52		0.53		0.58		0.59		0.55		*0.24*		*0.14*	0.73	0.85	0.93	1.09
RMSE	0.45	0.31		0.73		0.72		0.73		0.76		0.77		0.74		*0.49*		*0.37*	0.85	0.92	0.96	1.05
MAE	0.36	0.23		0.58		0.57		0.58		0.60		0.60		0.58		*0.38*		*0.30*	0.66	0.73	0.75	0.83
Effect size of prediction																						
$\hspace{1em}\beta$ (95% CI)	0.13 (− 0.01, 0.27)	0.11 (− 0.04, 0.25)		0.23 (− 0.11, 0.58)		0.11 (− 0.31, 0.54)		0.22 (− 0.10, 0.53)		0.05 (− 0.26, 0.37)		0.10 (− 0.16, 0.36)		0.04 (− 0.23, 0.31)		0.17 (0.01, 0.33)		0.13 (− 0.03, 0.30)	0.16 (− 0.27, 0.59)	0.06(− 0.45, 0.57)	0.00 (− 0.30, 0.30)	− 0.07 (− 0.39, 0.26)

*SD*, standard deviation; *CI*, confidence interval; base model is multiple linear regression model adjusted for sex, age group, medication, living area, distance from the pollution source, urinary cotinine level, and duration of occupational exposure; *ENET*, elastic net; *AENET*, adaptive elastic net; *WQS*, weighted quantile sum regression; *BKMR*, Bayesian kernel machine regression; *BART*, Bayesian additive regression tree; *SL*, super learner; *MSPE*, mean-square-prediction error; *RMSE*, root-mean-square error; *MAE*, mean absolute error

Original was the total population dataset (*n* = 256), and the test was sampled using stratified sampling from the original dataset, with consideration of all covariates and a sampling ratio of 80% (n = 199)

Kidney damage markers were base-10 log-transformed and scaled, and metabolites were scaled. GFR = 141 × min (S_cr_/κ, 1)^α^ × max (S_cr_/κ, 1)^−1.209^ × 0.993^Age^ × 1.018 [if female], where S_cr_ is serum creatinine in mg/dL, *κ* is 0.7 for females and 0.9 for males, *α* is − 0.329 for females and − 0.411 for males, min indicates the minimum of Scr/κ or 1, and max indicates the maximum of Scr/κ or 1

Most of the concentrations of urinary metabolites were higher in residents with a history of OCE (OCE group) compared to those without (non-OCE group), especially for MHA (*p* = 0.038). However, Hg was statistically lower in the OCE group than that in the non-OCE group (Table A4).

**Fig. 3:**
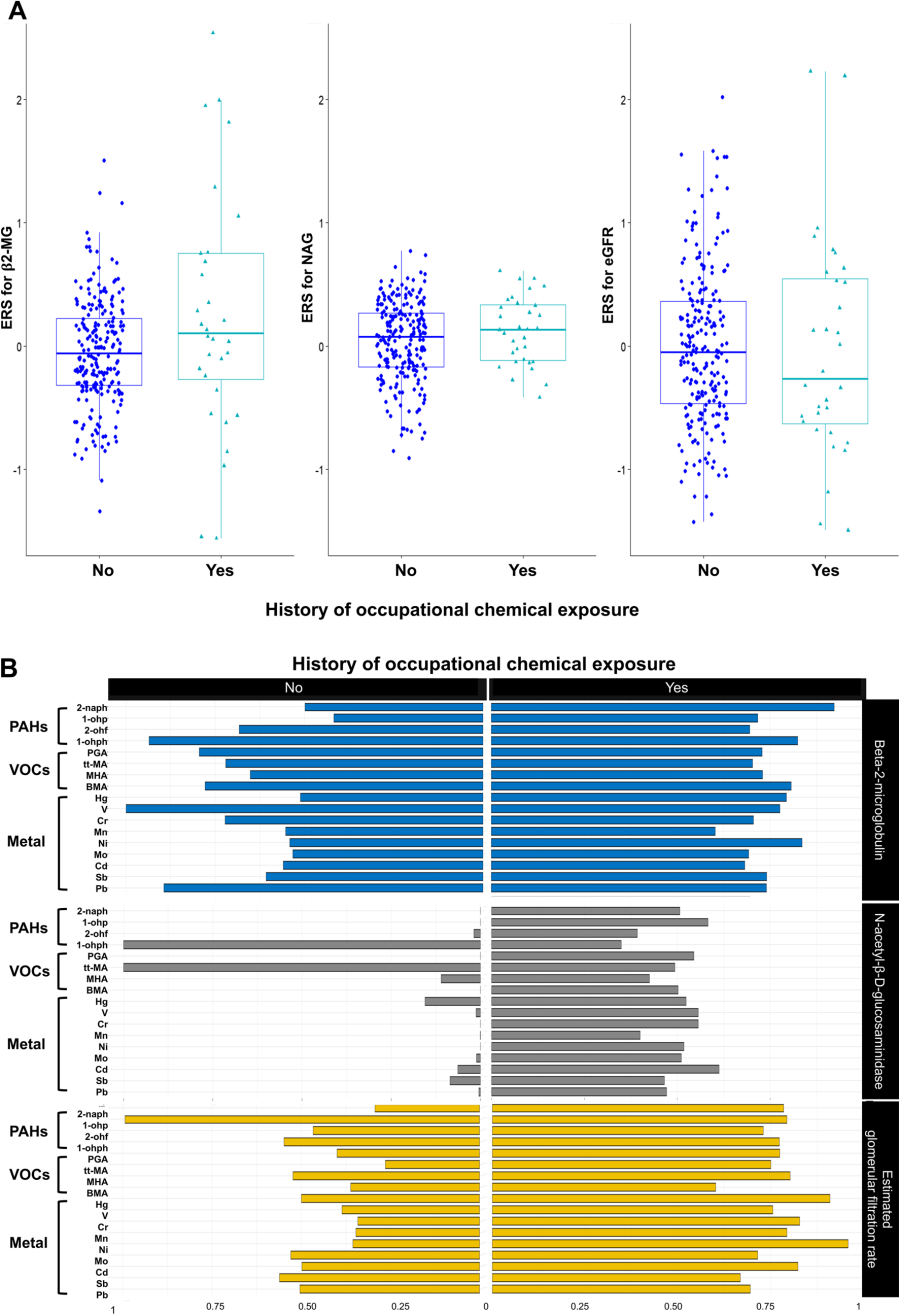
Distribution of ERS (**A**) and posterior inclusion probability of metabolites according to the history of occupational exposure (**B**). β2-MG, beta-2-microglobulin; NAG, N-acetylglucosaminidase; eGFR, estimate glomerular filtration rate; PAHs, polycyclic aromatic hydrocarbons; 2-naphthol (2-naph), 1-hydroxypyrene (1-ohp), 2-hydroxyfluorene (2-ohf), and 1-hydroxyphenanthrene (1-ohph); VOCs, volatile organic compounds; PGA, phosphoglyceric acid; t,t-MA, trans, trans-muconic acid; MHA, methylhippuric acid; BMA, benzylmercapturic acid

Figure 3 and Table A5 show the distribution of ERS based on the BKMR and PIP according to the history of OCE and kidney markers. In the OCE group, the ERS value exhibited an increasing trend compared to the non-OCE group for all KD markers, although it was not statistically significant (*p* values: β2-MG 0.074, NAG 0.21, and eGFR 0.52). For β2-MG, urinary V (PIP = 1.00) had the best effect in the non-OCE group and 2-naph (PIP = 0.92) in the OCE group. For NAG, 1-ohph and t,t-MA (PIP = 1.00) had the best effect in the non-OCE group and urinary Cd (PIP = 0.61) in the OCE group. Finally, for eGFR, 1-ohf (PIP = 0.99) had the best effect in the non-OCE group and urinary Ni (PIP = 0.96) in the OCE group.

Model fitness was higher using the ERS compared to the base model, which was adjusted for all covariates in multiple logistic regression. For the non-OCE group, the AUC values were 0.90, 0.87, and 0.91 in the ERS model and 0.83, 0.48, and 0.53 in the base model for β2-MG, NAG, and eGFR, respectively; for the OCE group, the AUC values were > 0.99, 0.98, and > 0.99 in the ERS model and > 0.99, 0.55, and 0.66 in the base model for β2-MG, NAG, and eGFR, respectively. The risks were higher in the OCE group compared to those in the non-OCE group. The OR (95% CI) for the ERS was 2.97 (2.19, 4.02) and 6.43 (2.85, 14.5) for β2-MG, 1.37 (1.01, 1.86) and 4.16 (1.85, 9.39) for NAG, and 4.57 (3.37, 6.19) and 6.44 (2.85, 14.5) for eGFR in the non-OCE and OCE group, respectively (Table 3).

**Table 3:** Odds ratio (OR) and 95% confidence interval (CI) of risk for kidney damage according to each environmental risk score (ERS) of urinary metabolites for biomarkers

History of occupational chemical exposure (OCE)							
Non-OCE (*n* = 224)				OCE (*n* = 32)			
OR (95% CI)		AUC	SPE/SEN (cut-off)	OR (95% CI)	AUC	SPE/SEN (cutoff)	
**β2-Microglobulin (β2-MG) > 300 µg/L (case, non-OCE 44; OCE 11)**							
**Base**	**NA**	**0.81**	**0.82/0.69 (NA)**	**NA**	** > 0.99**	** > 0.99/ > 0.99 (NA)**	
Kidney biomarker using ERS of urinary metabolites							
**β2-MG**	**2.97 (2.19, 4.02)**	**0.90**	**0.93/0.72 (0.10)**	**6.43 (2.85, 14.5)**	** > 0.99**	** > 0.99/ > 0.99 (1.00)**	
NAG	1.16 (0.85, 1.57)	0.60	0.39/0.84 (0.41)	2.50 (1.11, 5.64)	0.74	> 0.99/0.62 (0.08)	
eGFR	1.23 (0.91, 1.66)	0.57	0.43/0.73 (0.38)	2.06 (0.91, 4.65)	0.68	0.91/0.57 (− 0.64)	
**N-acetylglucosaminidase (NAG) > 11.5 IU/L (case, non-OCE 32; OCE 7)**							
**Base**	**NA**	**0.47**	**0.30/0.76 (NA)**	**NA**	**0.59**	**0.27/0.95 (NA)**	
Kidney biomarker using ERS of urinary metabolites							
β2-MG	1.17 (0.86, 1.58)	0.53	0.16/0.97 (1.22)	1.29 (0.57, 2.91)	0.62	0.71/0.64 (0.36)	
** NAG**	**1.37 (1.01, 1.86)**	**0.87**	**0.91/0.70 (0.21)**	**4.16 (1.85, 9.39)**	**0.98**	** > 0.99/0.92 (0.40)**	
eGFR	0.95 (0.70, 1.29)	0.52	0.53/0.60 (− 0.25)	0.78 (0.34, 1.75)	0.53	0.86/0.40 (− 0.46)	
**Estimated glomerular rate (eGFR) < 90 (case, non-OCE 141; OCE 18)**							
**Base**	**NA**	**0.54**	**0.61/0.49 (NA)**	**NA**	**0.65**	**0.91/0.57 (NA)**	
Kidney biomarker using ERS of urinary metabolites							
β2-MG	1.19 (0.88, 1.61)	0.55	0.80/0.35 (-0.55)	1.62 (0.72, 3.66)	0.65	0.72/0.79 (0.23)	
NAG	1.30 (0.96, 1.76)	0.55	0.83/0.30 (-0.24)	2.80 (1.24, 6.31)	0.74	0.94/0.64 (-0.23)	
**eGFR**	**4.57 (3.37, 6.19)**	**0.91**	**0.82/0.88 (− 0.18)**	**6.44 (2.85, 14.51)**	** > 0.99**	** > 0.99/ > 0.99 (− 0.49)**	

*AUC*, area under the receiver operating characteristic (ROC) curve; *SPE*, specificity; *SEN*, sensitivity; *Cutoff*, cut-off value of ERS

GFR = 141 × min (S_cr_/κ, 1)^α^ × max (S_cr_/κ, 1)^−1.209^ × 0.993^Age^ × 1.018 [if female], where S_cr_ is serum creatinine in mg/dL, *κ* is 0.7 for females and 0.9 for males, *α* is − 0.329 for females and − 0.411 for males, min indicates the minimum of Scr/κ or 1, and max indicates the maximum of Scr/κ or 1

The ERS was estimated using the Bayesian kernel machine regression (BKMR) adjusted for sex, age group, medication, living area, distance from the pollution source, urinary cotinine level, and duration of occupational exposure

OR and 95% CI of the base model were estimated using multiple logistic regression model adjusted for the same covariates with BKMR, and those of the ERS model were estimated using a simple logistic regression model

## Discussion

We found that the pollutant mixture model was effective in assessing health effects, exhibiting stability and excellent performance. The main composition of the mixture and health effects varied depending on the history of OCE. Furthermore, the OCE group was more vulnerable to environmental exposure compared to the non-OCE group.

The ERS of the pollutant mixture demonstrated stability and outperformed. Similar results have been observed in a previous study (Fu et al. 2022), which found that the association between ERS with heart rate and metabolic syndrome was stronger for MP compared to that of a single pollutant. Especially, BKMR is a successful statistical method for considering complex mixtures (Bobb et al. 2015). Thus, we propose that the predictability of KD is enhanced by the MP model.

Notably, the effects of urinary metals in the mixture differed depending on the KD markers. In the case of NAG, the effect of metals was small, whereas it was increased in β2-MG and significantly increased in eGFR. These differences may be owing to the mechanism by which metals affect AKI and CKD as they are rapidly cleared from the blood and sequestered in many tissues (Lentini et al. 2017). The first zone of the proximal tubule is the main site of reabsorption, and the luminal fluid can have bound and free forms in the early proximal tubule. AKI is induced by the ionized form that produces direct cellular toxicity, cellular membrane disruption, and uncoupling of the mitochondrial respiration pathway, whereas in CKD, the bound form can accumulate and cause chronic inflammation, fibrosis, and renal failure in the early proximal tubule (Lentini et al. 2017). In particular, β2-MG and NAG, as the indicators of AKI, exhibited a difference between glomerular transit and reabsorption (Ha et al. 1992). NAG measured the degree of damage to proximal tubular cells without passing through the glomerulus, whereas β2-MG first passed through the glomerulus and was then reabsorbed in the proximal tubular cells. Therefore, measuring β2-MG may be a more sensitive diagnostic method than measuring NAG (Ha et al. 1992). The reason for this is that the heavy metal exposure may have a low effect size on NAG depending on the cumulative exposure and reabsorption. However, if reabsorption damage continues, it may become significant in β2-MG, and if the exposure is chronic, it may ultimately affect eGFR.

The present study found that urinary Ni, Hg, and Cd and PAHs had a strong effect on KD in the OCE and non-OCE groups, respectively. Urinary Hg and Cd were the main exposure metabolites in the study areas (Kim et al. 2016; Kwon 2011). Ni is widely used in stainless steel manufacturing, electroplating, foundry applications, printing inks, and prostheses manufacturing, which causes its internal levels to be significantly higher among exposed workers (Tavares et al. 2022). PAHs affect the kidneys by increasing reactive oxygen species, resulting in an increase in oxidative stress, inducing apoptotic signals (Farzan et al. 2016), ultimately causing AKI and CKD (Lentini et al. 2017).

In the present study, there was no difference in the levels of ERS according to the OCE, but the OCE group showed a higher OR for kidney damage compared to the non-OCE group. This is because the non-OCE group was also affected by the mixture of pollutants. The study areas are vulnerable areas in Korea, and their residents have been reported to be exposed to environmental pollutants in the long term regardless of their history of OCE. A survey for health effects conducted in Goseong and Sangchon had reported that the residents living around abandoned mines had higher Cd concentration (3.30 µg/L in blood and 2.10 µg/g Crea. in urine) compared to the control group (2.24 µg/L in blood and 1.53 µg/g Crea. in urine) (Kwon 2011). Similarly, a previous study in Janghang reported that residents living 1–2 km from the smelter had higher concentrations of urinary As, blood Pb, and blood and urinary Cd compared to control groups, with a geometric mean of 9.25 and 8.36 µg/g Crea. 4.16 and 3.13 µg/dL, 2.59 and 1.71 µg/L, and 2.71 and 1.66 µg/g Crea., in the exposed areas and control group, respectively (Kim et al. 2016). Although it is challenging to generalize our results, we did successfully assess the impact of MP on health and selected the optimal model among various machine learning methods and analyzed the stratification history of OCE.

We showed that the OCE group was more vulnerable to the exposure to environmental pollutants compared to the non-OCE group. The prevalence of diabetes in the OCE group (31.2%) was higher than in the non-OCE group (16.1%) but not significant (Table A6), and the effect was adjusted as taking medication. Workers in the chemical industry are widely exposed to hazardous chemicals. According to the US National Health and Nutrition Examination Survey, workers in chemical industries had the highest exposure, followed by highly exposed residents and the general population (International Labour Organization 2021). A study on welders identified Hg, Cr, and Mn from metal mixtures as potential hazards (Zhang et al. 2017). Previous epidemiological studies have focused on constructing the ERS for the general population or specific groups (Park et al. 2017; Sun et al. 2013), and hence, studies based on the history of OCE are limited. Thus, the findings of the present study are meaningful since the model fitness was good despite the small size of the OCE group. We emphasize the necessity for further environmental exposure research in the OCE group.

This study had several limitations. First, the sample size was small and, therefore, did not represent nationwide coverage; moreover, it may not accurately reflect the characteristics of residents in vulnerable areas. Therefore, we performed a sensitivity analysis using data from the Korean National Environmental Health Survey (KNEHS), which provides chemical exposure information for the general population (NIER, 2018). However, KNEHS only measured four heavy metals (blood Pb and Hg and urinary Hg and Cd), which may not be appropriate for comparison with our study on various metals. For this reason, we measured exposure using biomarkers and adjusted the areas in the analysis of the models. Additionally, to overcome this limitation in the future, we plan to collect samples from vulnerable and control areas over a longer term. Second, there may be unmeasured confounders in our risk estimates due to the limitations of using the cross-sectional questionnaire. Although we investigated various diseases, many missing values were present in the responses to the questionnaire on disease history. Furthermore, since chemical exposure may differ based on occupation, it was challenging to consider each case in one model. To overcome this limitation, we controlled all possible confounders using the limited data available. For example, we used medication instead of the questionnaire on disease and used the total occupational exposure period. We also applied a dichotomous variable for the urinary biomarkers. Third, we did not consider the factor of career change for the participants. Some studies examining retirees have observed a declining pattern in chemical exposure levels, alongside non-significant impacts on microcirculation abnormalities and bone health (Li et al. 2020; Lopez et al. 2013; Platts et al. 2013). However, due to the limitations of the survey questions, it was not possible to trace the history of occupational exposure. Therefore, we focused on the cumulative effects of exposure. Fourth, we obtained the occupational history via only a self-reported survey. Finally, information bias could be present in the data, such as interviewer, measurement, and/or recall bias. Therefore, bias may have caused differential misclassification between the exposed and control areas. Hence, we consider the biomarkers as the exposed factors sufficient to overcome these misclassifications. Finally, we only analyzed urine samples, which is appropriate as they are considered indicative of long-term exposure (Vacchi-Suzzi et al. 2016).

Despite these limitations, this study successfully constructed the ERS of MPs, including metals, PAHs, and VOCs, for KD, and considered the history of OCE in environmentally vulnerable residents. This is unique for the field of environmental epidemiology because similar studies have often considered only the association between a single pollutant and health outcome.

## Conclusion

We identified the most appropriate ERS of harmful metabolites for the exposure to MP that cause KD based on a history of OCE in environmentally vulnerable areas in Korea. In addition, we established that the OCE group has a higher risk of environmental exposure to MP that cause KD.

### Supplementary Information

Below is the link to the electronic supplementary material.

Supplementary file1 (DOCX 1056 KB)

## Data Availability

Due to privacy and ethical concerns, neither the data nor the source of the data can be made available.

## Abbreviations

1-ohp: 1-Hydroxypyrene
1-ohph: 1-Hydroxyphenanthrene
2-naph: 2-Naphthol
2-ohf: 2-Hydroxyfluorene
AENET: Adaptive elastic net
AKI: Acute kidney injury
AUC: Area under the ROC curve
BART: Bayesian additive regression tree
BKMR: Bayesian kernel machine regression
BMA: Benzyl mercapturic acid
β2-MG: Beta-2-microglobulin
Cd: Cadmium
CI: Confidence interval
CKD: Chronic kidney disease
Cr: Chromium
eGFR: Estimated glomerular filtration rate
ENET: Linear model elastic net
ERS: Environmental risk score
FROM: Forensic Research via Omics Markers
GM: Geometric mean
Hg: Mercury
KD: Kidney damage
KNEHS: Korean National Environmental Health Survey
LOD: Limit of detection
MAE: Mean absolute error
MHA: Methyl hippuric acid
Mn: Manganese
Mo: Molybdenum
MP: Multiple pollutants
MSPE: Mean-squared-prediction error
NAG: N-acetyl-β-D-glucosaminidase
Ni: Nickel
OCE: Occupational chemical exposure
OR: Odds ratio
PAHs: Polycyclic aromatic hydrocarbons
Pb: Lead
PGA: Phosphoglyceric acid
PIP: Posterior inclusion probability
RMSE: Root-mean-square error
ROC: Receiver operating characteristic
Sb: Antimony
SL: Super learner
t,t-MA: Transtrans-muconic acid
V: Vanadium
VI: Variable importance
VOCs: Volatile organic compounds
WQS: Weighted quantile sum regression
