# Supplementary material for: Environmental risk score of multiple pollutants for kidney damage among residents in vulnerable areas by occupational chemical exposure in Korea
Source: Environ Sci Pollut Res Int. 2024 May 14;31(24):35938–51. doi: 10.1007/s11356-024-33567-5 (PMC11136836; doi:10.1007/s11356-024-33567-5)
Supplement: Supplementary file 1 — Supplementary file1 (DOCX 1056 KB) [file 11356_2024_33567_MOESM1_ESM.docx]

Environmental risk score of multiple pollutants for kidney damage among residents in vulnerable areas by occupational chemical exposure in Korea

Supplementary Materials

Table A1. List of R packages used for assessing environmental risk score

| Model | Package name |
| --- | --- |
|  |  |
| Elastic net | cv.glmnet and caret |
| Adaptive elastic net | cv.gcdnet |
| Weighted quantile sum regression | gWQS |
| Bayesian kernel machine regression | bkmr |
| Bayesian additive regression tree | Bayes Tree |
| Super learner | e1071  bartMachineCV  CV.SuperLearner  gam  ipred  missForest  itertools  iterators  xgboost |


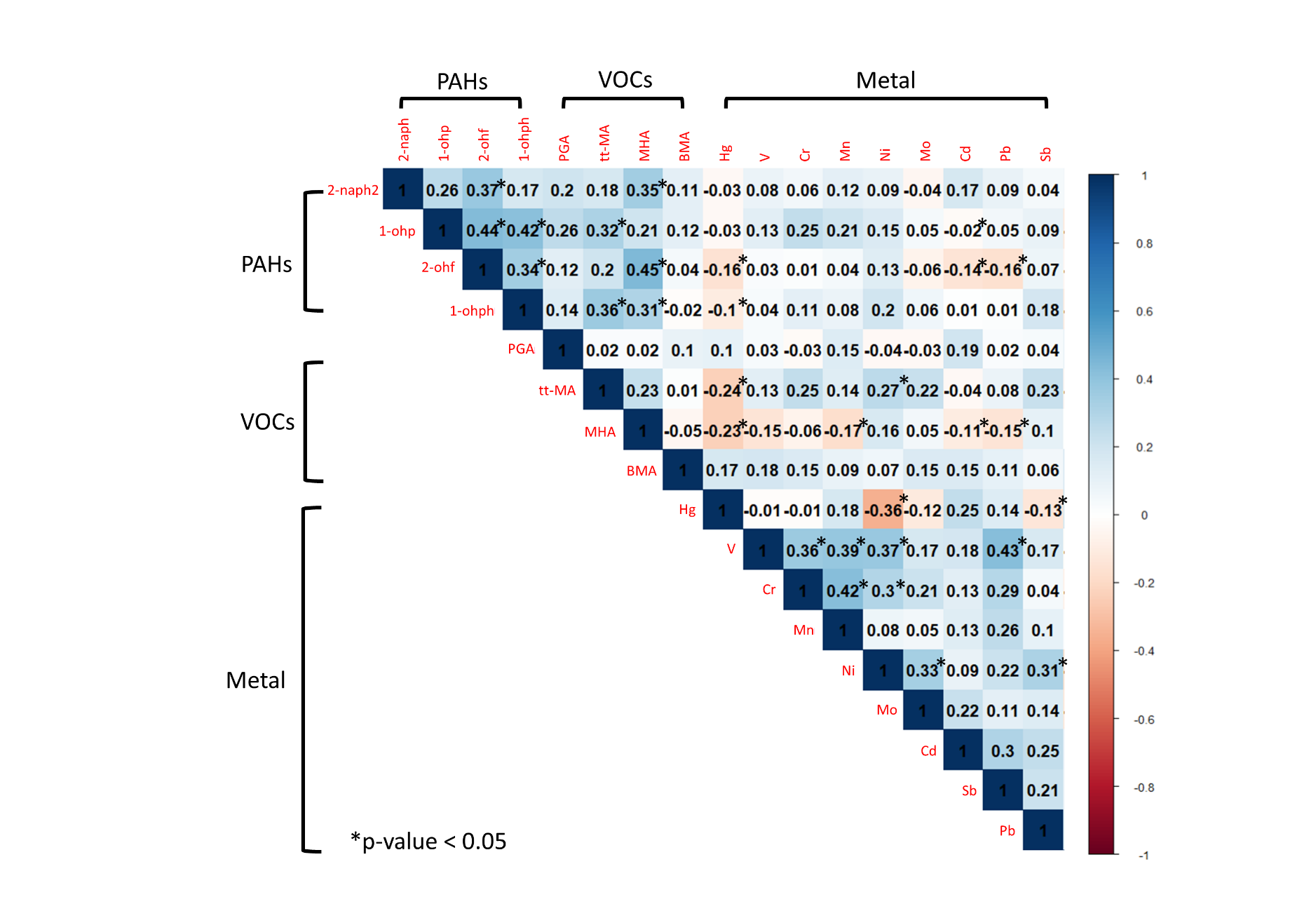


Fig A1 Correlation plot of urinary concentrations of metabolites for environmental hazard materials. PAHs, polycyclic aromatic hydrocarbons; 2-naph, 2-naphthol; 1-ohp, 1-hydroxypyrene; 2-ohf, 2-hydroxyfluorene; 1-ohph, 1-hydroxyphenanthrene; VOCs, volatile organic compounds; PGA, phosphoglyceric acid, t,t-MA, trans, trans-muconic acid, MHA, methylhippuric acid; BMA, benzylmercapturic acid; β2-MG, beta-2-microglobulin; NAG, N-acetylglucosaminidase; eGFR, estimate glomerular filtration rate

Table A2. General characteristics for original and test datasets

|  | Original data | Test data | *p* value^a^ |
| --- | --- | --- | --- |
|  | N (%) | N (%) |  |
| All | 256 (100) | 199 (100) |  |
| Gender |  |  | > 0.999 |
| Male | 99 (38.67) | 77 (38.69) |  |
| Female | 157 (61.33) | 122 (61.31) |  |
| Age (years) |  |  | 0.996 |
| < 65 | 69 (26.95) | 53 (26.63) |  |
| 65–74 | 94 (36.72) | 73 (36.68) |  |
| 75+ | 93 (36.33) | 73 (36.68) |  |
| Co-morbidity (Hypertension or Diabetes) |  |  | 0.446 |
| No | 117 (45.70) | 99 (49.75) |  |
| Yes | 139 (54.30) | 100 (50.25) |  |
| Medication for any disease |  |  | 0.870 |
| No | 49 (19.14) | 36 (18.09) |  |
| Yes | 207 (80.86) | 163 (81.91) |  |
| Area^b^ |  |  | > 0.999 |
| Gimhae | 32 (12.50) | 25 (12.56) |  |
| Goseong | 68 (26.56) | 53 (26.63) |  |
| Sangchon | 63 (24.61) | 49 (24.62) |  |
| Janghang | 93 (36.33) | 72 (36.18) |  |
| Period of residence (years) |  |  | 0.933 |
| T1 (<12.92) | 85 (33.20) | 68 (34.17) |  |
| T2 (12.92–< 50.00) | 80 (31.25) | 59 (29.65) |  |
| T3 (50.00–93.00) | 91 (35.55) | 72 (36.18) |  |
| Urinary cotinine level (μg/g Crea.) |  |  |  |
| T1 (< 2.09) | 85 (33.20) | 61 (30.65) |  |
| T2 (2.09–< 6.97) | 85 (33.20) | 68 (34.17) |  |
| T3 (6.97-3851.99) | 86 (33.59) | 70 (35.18) |  |
| History of occupational chemical exposure |  |  | 0.844 |
| No | 224 (87.50) | 174 (87.44) |  |
| Yes | 32 (12.50) | 25 (12.56) |  |
| Period of occupational chemical exposure (years) |  |  | > 0.999 |
| None | 224 (87.50) | 174 (87.44) |  |
| <4.5 | 16 (6.3) | 9 (4.52) |  |
| 4.5–≤60 | 16 (6.3) | 16 (8.04) |  |
| Metabolites in urine (μg/g Crea.), GM (95% CI) |  |  |  |
| Metals |  |  |  |
| Hg | 0.65 (0.58, 0.72) | 0.67 (0.59, 0.76) | 0.741 |
| V | 0.34 (0.31, 0.36) | 0.34 (0.32, 0.37) | 0.708 |
| Cr | 0.32 (0.29, 0.36) | 0.32 (0.28, 0.36) | 0.884 |
| Mn | 0.26 (0.23, 0.29) | 0.26 (0.23, 0.30) | 0.980 |
| Ni | 3.66 (3.36, 4.00) | 3.58 (3.24, 3.96) | 0.730 |
| Mo | 96.39 (89.23, 104.11) | 90.75 (83.41, 98.73) | 0.301 |
| Cd | 1.73 (1.58, 1.89) | 1.77 (1.60, 1.97) | 0.716 |
| Sb | 0.10 (0.09, 0.11) | 0.10 (0.09, 0.11) | 0.840 |
| Pb | 0.80 (0.72, 0.90) | 0.79 (0.69, 0.89) | 0.799 |
| Polycyclic Aromatic Hydrocarbons |  |  |  |
| 2-naphthol | 3.68 (3.17, 4.27) | 3.87 (3.25, 4.60) | 0.667 |
| 1-Hydroxypyrene | 0.13 (0.11, 0.15) | 0.13 (0.11, 0.15) | 0.818 |
| 2-Hydroxyfluorene | 0.16 (0.14, 0.18) | 0.16 (0.14, 0.19) | 0.862 |
| 1-Hydroxyphenanthrene | 0.08 (0.07, 0.09) | 0.08 (0.07, 0.09) | 0.900 |
| Volatile Organic Compounds |  |  |  |
| Phosphoglyceric acid | 109.54 (96.15, 124.79) | 114.33 (98.95, 132.10) | 0.666 |
| Trans, trans-muconic acid | 65.44 (59.01, 72.57) | 64.48 (57.39, 72.44) | 0.851 |
| Methylhippuric acid | 70.59 (60.70, 82.11) | 75.81 (63.61, 90.35) | 0.543 |
| Benzylmercapturic | 11.74 (10.43, 13.22) | 11.63 (10.17, 13.30) | 0.915 |

Testing dataset was sampled data from the original dataset using systematic sampling.

GM, geometric mean; CI, confidence interval

^a^*p* value estimated using chi-squared test or t-test

^b^The pollution source of Goseong and Sangchon are abandoned metal mines, and that of Janghang is smelter area. The participants in Goseong, Sangchon, and Janghang lived within distances of 1.18 to 3.76 km, 4.09 to 5.63 km, and 1.33 to 23.60 km from the source of pollution.
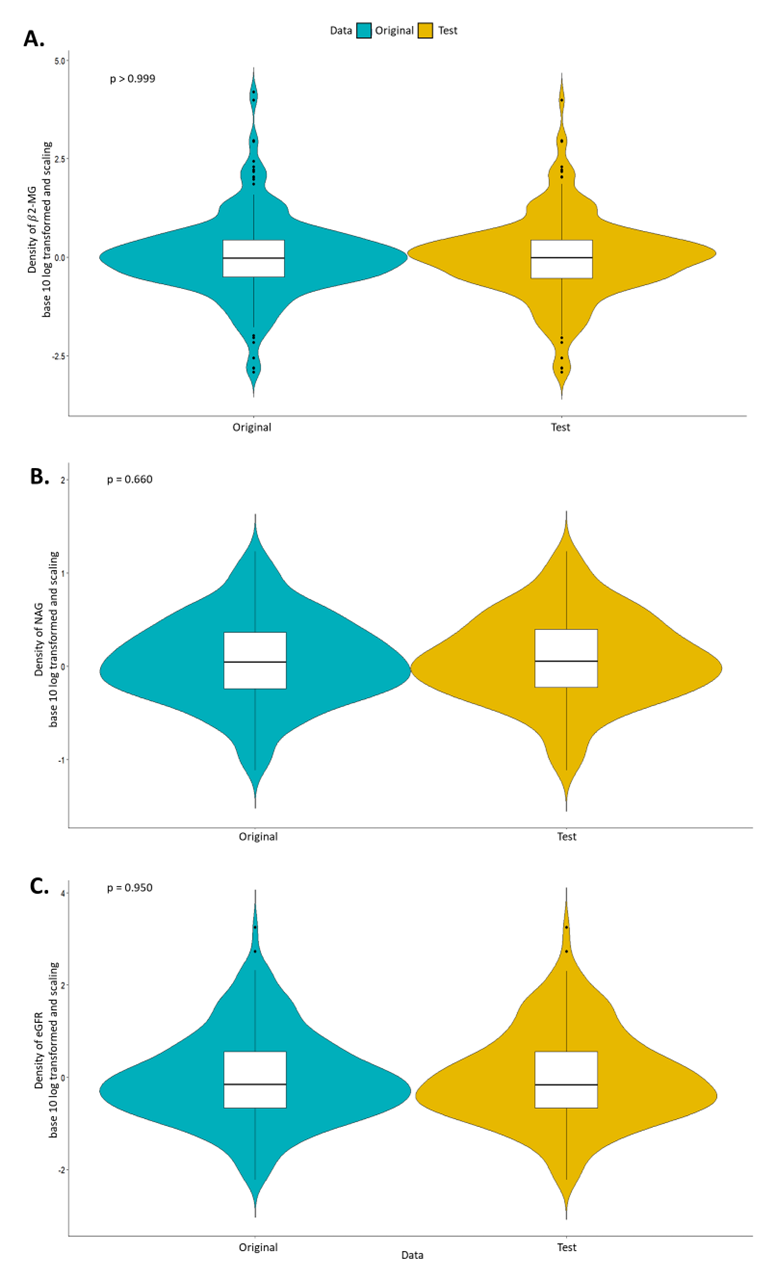


Fig. A2 Density of kidney damage marker according to the data on β2-microglobulin (A), N-acetyl glucosaminidase (B), and estimated glomerular filtration rate (eGFR) (C). eGFR = 141 × min (S_cr_/κ, 1)^α^ × max(S_cr_/κ, 1)^-1.209^ × 0.993^Age^ × 1.018 [if female], where S_cr_ is serum creatinine in mg/dL, κ is 0.7 for females and 0.9 for males, α is −0.329 for females and −0.411 for males, min indicates the minimum of Scr/κ or 1, and max indicates the maximum of Scr/κ or 1. Original was total population dataset (n = 256), and test was sampled using stratified sampling from the original dataset, with consideration of all covariates and a sampling ratio of 80% (n = 199). *p* value was estimated using t-test.

Table A3. Comparison of the distribution and performance of environmental risk score (ERS) of urinary metabolites for biomarkers by kidney damage markers (Crude model for Table 2)

|  | Model | | | | | | | | | | | | | |
| --- | --- | --- | --- | --- | --- | --- | --- | --- | --- | --- | --- | --- | --- | --- |
|  | Base | | ENET | | AENET | | WQS | | BKMR | | BART | | SL | |
| Data | Original | Test | Original | Test | Original | Test | Original | Test | Original | Test | Original | Test | Original | Test |
|  | β2-Microglobulin (μg/dL) | | | | | | | | | | | | | |
| Distribution of ERS | | | | | | | | | | | | | | |
| Mean (SD) | 0.01 (0.83) | −0.02 (0.92) | 0.01 (0.21) | −0.02 (0.14) | 0.01 (0.18) | −0.02 (0.17) | 0.01 (0.34) | −0.02 (0.38) | −0.01 (0.64) | −0.02 (0.62) | 0.01 (0.45) | −0.01 (0.31) | 0.00 (0.44) | −0.01 (0.49) |
| Range (Min, Max) | (−2.46, 3.05) | (−2.76, 3.34) | (−0.53, 0.80) | (−0.38, 0.39) | (−0.45, 0.77) | (−0.43, 0.75) | (−0.72, 0.75) | (−0.79, 0.80) | (−1.78, 2.67) | (−1.74, 1.93) | (−0.97, 1.53) | (−0.70, 0.75) | (−1.21, 1.14) | (−1.50, 1.26) |
| Risk prediction performance | | | | | | | | | | | | | | |
| R^2^ | 0.71 | 0.83 | 0.22 | 0.14 | 0.16 | 0.15 | 0.11 | 0.14 | *0.91* | *0.83* | 0.56 | 0.41 | 0.09 | 0.13 |
| MSPE | 0.28 | 0.18 | 0.83 | 0.93 | 0.87 | 0.92 | 0.87 | 0.88 | *0.19* | *0.27* | 0.52 | 0.71 | 0.91 | 0.91 |
| RMSE | 0.53 | 0.42 | 0.91 | 0.97 | 0.93 | 0.96 | 0.93 | 0.94 | *0.43* | *0.52* | 0.72 | 0.84 | 0.95 | 0.95 |
| MAE | 0.42 | 0.33 | 0.65 | 0.69 | 0.66 | 0.69 | 0.68 | 0.70 | *0.31* | *0.38* | 0.52 | 0.60 | 0.70 | 0.70 |
|  | N-acetylglucosaminidase (IU/L) | | | | | | | | | | | | | |
| Distribution of ERS | | | | | | | | | | | | | | |
| Mean (SD) | 0.06 (0.37) | 0.08 (0.41) | 0.06 (0.08) | 0.08 (0.07) | 0.06 (0.05) | 0.08 (0.09) | 0.06 (0.19) | 0.08 (0.21) | 0.06 (0.40) | 0.07 (0.34) | 0.06 (0.12) | 0.08 (0.18) | 0.04 (0.26) | 0.06 (0.30) |
| Range (Min, Max) | (−1.06, 1.15) | (−1.23, 1.09) | (−0.14, 0.38) | (−0.10, 0.33) | (−0.08, 0.27) | (−0.10, 0.44) | (−0.41, 0.55) | (−0.44, 0.68) | (−1.00, 1.10) | (−0.80, 0.90) | (−0.22, 0.37) | (−0.33, 0.56) | (−0.64, 0.80) | (−0.67, 1.08) |
| Risk prediction performance | | | | | | | | | | | | | | |
| R^2^ | 0.68 | 0.80 | 0.19 | 0.19 | 0.14 | 0.20 | 0.17 | 0.20 | *1.00* | *0.97* | 0.30 | 0.46 | 0.07 | 0.13 |
| MSPE | 0.06 | 0.04 | 0.18 | 0.19 | 0.19 | 0.18 | 0.17 | 0.17 | *0.00* | *0.02* | 0.16 | 0.13 | 0.21 | 0.20 |
| RMSE | 0.25 | 0.20 | 0.42 | 0.43 | 0.43 | 0.43 | 0.41 | 0.41 | *0.05* | *0.14* | 0.40 | 0.36 | 0.45 | 0.45 |
| MAE | 0.20 | 0.16 | 0.33 | 0.34 | 0.34 | 0.33 | 0.32 | 0.32 | *0.04* | *0.11* | 0.31 | 0.28 | 0.35 | 0.35 |
|  | Estimated glomerular filtration rate (eGFR) | | | | | | | | | | | | | |
| Distribution of ERS | | | | | | | | | | | | | | |
| Mean (SD) | −0.02 (0.76) | −0.01 (0.87) | −0.02 (0.16) | −0.01 (0.20) | −0.02 (0.19) | −0.01 (0.12) | −0.02 (0.19) | −0.01 (0.22) | −0.02 (0.75) | −0.02 (0.42) | −0.02 (0.36) | 0.00 (0.21) | −0.02 (0.40) | −0.03 (0.35) |
| Range (Min, Max) | (−2.20, 2.49) | (−2.13, 2.79) | (−0.58, 0.91) | (−0.72, 1.06) | (−0.73, 1.16) | (−0.50, 0.96) | (−0.49, 0.51) | (−0.51, 0.57) | (−1.73, 2.58) | (−0.93, 1.21) | (−0.88, 0.97) | (−0.49, 0.53) | (−1.08, 1.38) | (−0.88, 1.14) |
| Risk prediction performance | | | | | | | | | | | | | | |
| R^2^ | 0.62 | 0.77 | 0.17 | 0.25 | 0.17 | 0.09 | 0.04 | 0.05 | 0.98 | 0.87 | 0.50 | 0.36 | 0.03 | 0.01 |
| MSPE | 0.36 | 0.22 | 0.83 | 0.82 | 0.82 | 0.92 | 0.89 | 0.93 | 0.06 | 0.38 | 0.57 | 0.77 | 0.95 | 1.02 |
| RMSE | 0.60 | 0.47 | 0.91 | 0.91 | 0.91 | 0.96 | 0.95 | 0.96 | 0.25 | 0.62 | 0.75 | 0.88 | 0.98 | 1.01 |
| MAE | 0.46 | 0.35 | 0.71 | 0.71 | 0.70 | 0.75 | 0.73 | 0.75 | 0.20 | 0.49 | 0.59 | 0.69 | 0.77 | 0.78 |

SD, standard deviation; CI, confidence interval; Base model is simple linear regression model; ENET, elastic net; AENET, adaptive elastic net; WQS, weighted quantile sum regression; BKMR, Bayesian kernel machine regression; BART, Bayesian additive regression tree; SL, super learner; MSPE, mean-square-prediction error; RMSE, root-mean-square error; MAE, mean absolute error.

Original was total population dataset (n=256), and test was sampled using stratified sampling from the original dataset, with consideration of all covariates and a sampling ratio of 80% (n=199)

Kidney damage markers were base-10 log transformed and scaled, and metabolites were scale.

eGFR = 141 × min (S_cr_/κ, 1)^α^ × max (S_cr_/κ, 1)^−1.209^ × 0.993^Age^ × 1.018 [if female], where S_cr_ is serum creatinine in mg/dL, κ is 0.7 for females and 0.9 for males, α is −0.329 for females and −0.411 for males, min indicates the minimum of Scr/κ or 1, and max indicates the maximum of Scr/κ or 1.


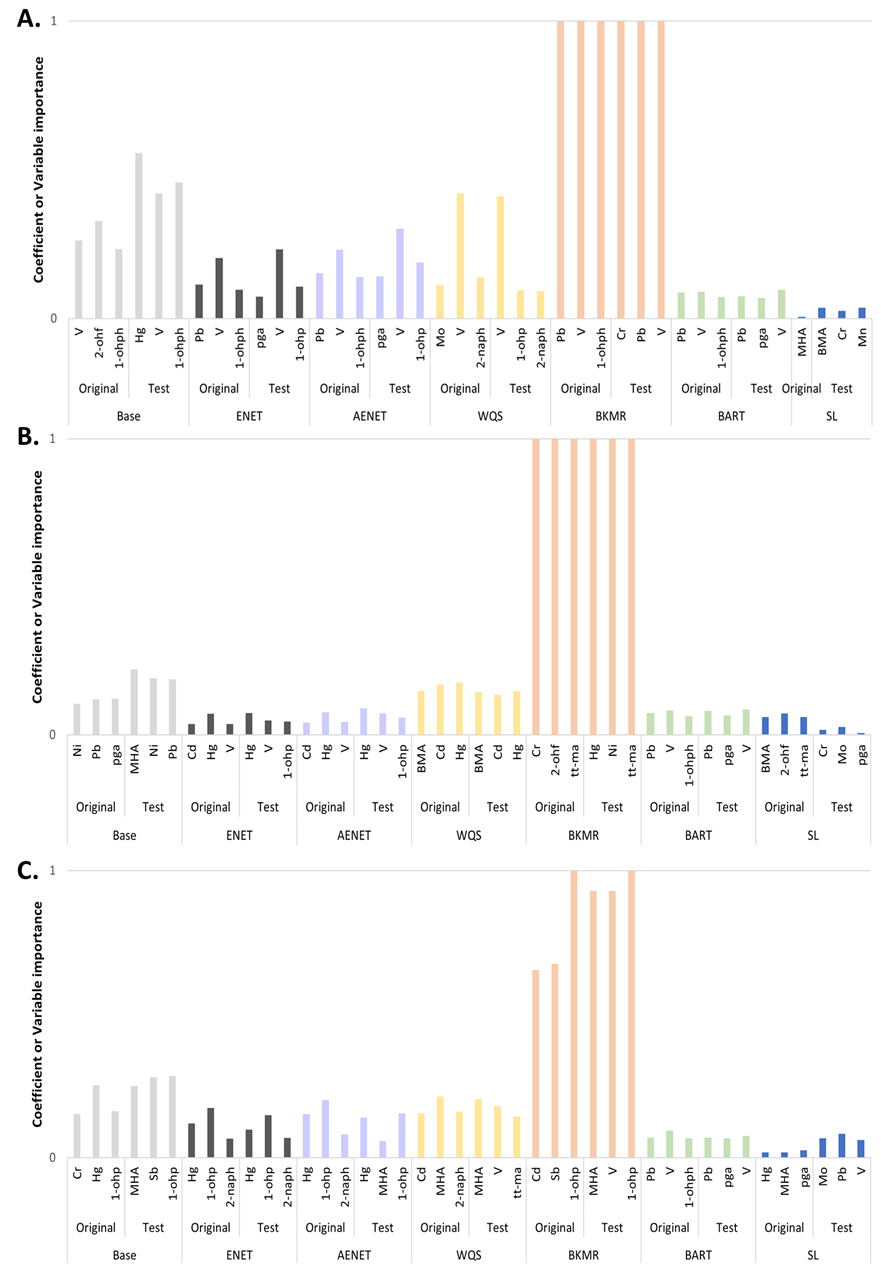


Fig. A3 Most effective metabolites according to ERS model and data types for each model: β2-microglobulin (A), N-acetyl glucosaminidase (B), and estimated glomerular filtration rate (eGFR) (C). Base model is multiple linear regression model; ENET, elastic net; AENET, adaptive elastic net; WQS, weighted quantile sum regression; BKMR, Bayesian kernel machine regression; BART, Bayesian additive regression tree; SL, super learner. Kidney damage markers were base-10 log transformed and scaled, and metabolites were scale. GFR = 141 × min (S_cr_/κ, 1)^α^ × max (S_cr_/κ, 1)^−1.209^ × 0.993^Age^ × 1.018 [if female], where S_cr_ is serum creatinine in mg/dL, κ is 0.7 for females and 0.9 for males, α is −0.329 for females and −0.411 for males , min indicates the minimum of Scr/κ or 1, and max indicates the maximum of Scr/κ or 1

Table A4. Distribution of concentration of urinary metabolites for environmental hazard materials.

| Concentration of urinary metabolites (μg/g Crea.) | All | History of occupational chemical exposure (OCE) | | | |
| --- | --- | --- | --- | --- | --- |
|  |  | Non-OCE (n=224) | | OCE (n=32) | p ^a^ |
|  | GM (95% CI) | GM (95% CI) | GM (95% CI) | |  |
| Metals |  |  |  | |  |
| Hg | 0.65 (0.58, 0.72) | 0.68 (0.61, 0.77) | 0.45 (0.34, 0.61) | | 0.013 |
| V | 0.34 (0.31, 0.36) | 0.33 (0.31, 0.36) | 0.36 (0.30, 0.44) | | 0.407 |
| Cr | 0.32 (0.29, 0.36) | 0.31 (0.28, 0.35) | 0.41 (0.28, 0.61) | | 0.117 |
| Mn | 0.26 (0.23, 0.29) | 0.27 (0.24, 0.30) | 0.21 (0.15, 0.28) | | 0.097 |
| Ni | 3.66 (3.36, 4.00) | 3.57 (3.24, 3.94) | 4.38 (3.59, 5.32) | | 0.134 |
| Mo | 96.39 (89.23, 104.11) | 97.64 (90.36, 105.50) | 88.06 (64.70, 119.85) | | 0.384 |
| Cd | 1.73 (1.58, 1.89) | 1.76 (1.60, 1.94) | 1.51 (1.12, 2.05) | | 0.272 |
| Sb | 0.10 (0.09, 0.11) | 0.10 (0.09, 0.11) | 0.09 (0.07, 0.12) | | 0.409 |
| Pb | 0.80 (0.72, 0.90) | 0.79 (0.70, 0.90) | 0.87 (0.61, 1.24) | | 0.589 |
| Polycyclic Aromatic Hydrocarbons |  |  |  | |  |
| 2-naphthol | 3.68 (3.17, 4.27) | 3.60 (3.08, 4.22) | 4.26 (2.61, 6.94) | | 0.470 |
| 1-Hydroxypyrene | 0.13 (0.11, 0.15) | 0.12 (0.11, 0.14) | 0.14 (0.09, 0.21) | | 0.574 |
| 2-Hydroxyfluorene | 0.16 (0.14, 0.18) | 0.15 (0.13, 0.17) | 0.22 (0.14, 0.34) | | 0.073 |
| 1-Hydroxyphenanthrene | 0.08 (0.07, 0.09) | 0.08 (0.07, 0.09) | 0.10 (0.08, 0.13) | | 0.173 |
| Volatile Organic Compounds |  |  |  | |  |
| Phosphoglyceric acid | 109.54 (96.15, 124.79) | 109.29 (95.11, 125.59) | 111.27 (74.82, 165.48) | | 0.929 |
| Trans, trans-muconic acid | 65.44 (59.01, 72.57) | 64.43 (57.71, 71.93) | 73.01 (53.33, 99.96) | | 0.432 |
| Methylhippuric acid | 70.59 (60.70, 82.11) | 66.48 (56.79, 77.83) | 107.48 (65.56, 176.18) | | 0.038 |
| Benzylmercapturic | 11.74 (10.43, 13.22) | 11.66 (10.32, 13.17) | 12.39 (8.04, 19.09) | | 0.737 |

GM, Geometric mean; CI, Confidence interval

^a^ p-value was estimated by t-test after log-transformation.

Table A5. Posterior inclusion probability (PIP) of environmental risk score (ERS) for kidney damage markers stratified with the history of occupational chemical exposure

| Urinary metabolites | Kidney damage markers | | | | | |
| --- | --- | --- | --- | --- | --- | --- |
|  | β2-Microglobulin | | N-acetylglucosaminidase | | eGFR | |
|  | History of occupational chemical exposure | | History of occupational chemical exposure | | History of occupational chemical exposure | |
|  | No | Yes | No | Yes | No | Yes |
|  | PIP^a^ | PIP^a^ | PIP^a^ | PIP^a^ | PIP^a^ | PIP^a^ |
| Metals |  |  |  |  |  |  |
| Hg | 0.51 | 0.79 | 0.16 | 0.52 | 0.50 | 0.91 |
| V | 1.00 | 0.78 | 0.01 | 0.56 | 0.39 | 0.76 |
| Cr | 0.72 | 0.70 | 0.00 | 0.56 | 0.34 | 0.83 |
| Mn | 0.55 | 0.60 | 0.00 | 0.40 | 0.35 | 0.79 |
| Ni | 0.54 | 0.84 | 0.00 | 0.52 | 0.36 | 0.96 |
| Mo | 0.53 | 0.69 | 0.01 | 0.51 | 0.53 | 0.72 |
| Cd | 0.56 | 0.68 | 0.06 | 0.61 | 0.50 | 0.82 |
| Sb | 0.61 | 0.74 | 0.09 | 0.47 | 0.56 | 0.67 |
| Pb | 0.89 | 0.74 | 0.00 | 0.47 | 0.50 | 0.70 |
| Polycyclic Aromatic Hydrocarbons | |  |  |  |  |  |
| 2-naphthol | 0.50 | 0.92 | 0.00 | 0.51 | 0.29 | 0.79 |
| 1-Hydroxypyrene | 0.42 | 0.72 | 0.00 | 0.58 | 0.99 | 0.79 |
| 2-Hydroxyfluorene | 0.68 | 0.69 | 0.02 | 0.39 | 0.47 | 0.73 |
| 1-Hydroxyphenanthrene | 0.94 | 0.82 | 1.00 | 0.35 | 0.55 | 0.77 |
| Volatile Organic Compounds |  |  |  |  |  |  |
| Phosphoglyceric acid | 0.80 | 0.73 | 0.00 | 0.54 | 0.40 | 0.77 |
| Trans, trans-muconic acid | 0.72 | 0.70 | 1.00 | 0.49 | 0.26 | 0.75 |
| Methylhippuric acid | 0.65 | 0.73 | 0.11 | 0.42 | 0.52 | 0.80 |
| Benzylmercapturic | 0.78 | 0.81 | 0.00 | 0.50 | 0.36 | 0.60 |

^a^Posterior inclusion probability estimated using Bayesian kernel machine regression (BKMR)

GFR = 141 × min (S_cr_/κ, 1)^α^ × max (S_cr_/κ, 1)^−1.209^ × 0.993^Age^ × 1.018 [if female], where S_cr_ is serum creatinine in mg/dL, κ is 0.7 for females and 0.9 for males, α is −0.329 for females and −0.411 for males, min indicates the minimum of Scr/κ or 1, and max indicates the maximum of Scr/κ or 1

Table A6. Prevalence of hypertension and diabetes according to the history occupational chemical exposure group

|  | History of occupational chemical exposure | | p-value |
| --- | --- | --- | --- |
|  | No (N = 224) | Yes (N = 32) |  |
| Disease (yes), N (%) |  |  |  |
| Hypertension | 108 (48.2) | 15 (46.9) | >0.999 |
| Diabetes | 35 (16.1) | 10 (31.2) | 0.065 |

p-value was estimated using chi-square test
